# Association between exposure to intimate partner violence and the nutritional status of women and children in Nigeria

**DOI:** 10.1371/journal.pone.0268462

**Published:** 2022-05-12

**Authors:** Abdul-Nasir Issah, Daudi Yeboah, Mary Rachael Kpordoxah, Michael Boah, Abraham Bangamsi Mahama

**Affiliations:** 1 Department of Health Services, Policy, Planning, Management and Economics, School of Public Health, University for Development Studies, Tamale, Ghana; 2 Department of Epidemiology, Biostatistics, and Disease Control, School of Public Health, University for Development Studies, Tamale, Ghana; 3 Department of Global and International Health, School of Public Health, University for Development Studies, Tamale, Ghana; 4 United Nations Children’s Fund (UNICEF) Nigeria Country Office, Garki, Abuja, Nigeria; National University of Sciences and Technology, PAKISTAN

## Abstract

**Background:**

Globally, intimate partner violence (IPV) epitomizes a greater proportion of the violence experienced by women, with more than a third of women (41.3%) in sub-Saharan Africa reporting IPV during their lifetime. This study examined the association between exposure to IPV and the nutritional status of women and their children in Nigeria.

**Methods:**

The study analyzed secondary data obtained from the 2018 Nigeria Demographic and Health Survey. Data on women’s lifetime experience of psychological, physical, and sexual IPV, as well as demographic and socioeconomic characteristics, were collected. We used regression models to determine the association between exposure to IPV and women and child nutrition indicators. A weighted sample of 4,391 women aged 15–49 years and 2,145 children 6–59 months were analyzed.

**Results:**

The lifetime experience of IPV in the study was 35.31% (95% CI: 33.35, 37.33), 30.43% (95% CI: 28.54, 32.38) experienced psychological IPV, 19.43% (95% CI: 17.79, 21.19) experienced physical IPV, and 6.03% (95% CI: 5.12, 7.09) experienced sexual IPV. After adjusting for a range of characteristics, maternal lifetime exposure to IPV was associated with underweight (ARRR = 0.63; 95% CI: 0.44, 0.91) and overweight/obesity (ARRR = 1.28; 95% CI: 1.04, 1.58). We also found that, children whose mothers experienced IPV were less likely to be underweight compared to their counterparts (ARRR = 0.69; 95% CI: 0.50, 0.96).

**Conclusions:**

Overall, IPV against women, particularly psychological, physical, and sexual IPV, is common in Nigeria and has an association with the nutritional status of affected women and their children. According to the study, women with a lifetime experience of IPV were more likely to be overweight. On the other hand, affected women’s children were less likely to be underweight. A far-reaching effort is required to curb IPV against women, particularly policies, programs, and laws are needed to protect women and children from the unfavourable effects of IPV to reduce the prevalence and impact of such violence.

## Introduction

Globally, violence by a husband or male intimate partner, whether physically, sexually, or psychologically, is the most widespread form of violence against women. According to the World Health Organization, intimate partner violence (IPV) refers to any behaviour by a current or former male intimate partner within the context of marriage, cohabitation, or any formal or informal union, that causes physical, sexual, or psychological harm [1].

In 2018, 27% of ever-married/partnered women aged 15–49 years have been subjected to physical and/or sexual violence in their lifetime, suggesting that 641 million and up to 753 million women of the reproductive age have experienced IPV at least once since they turned 15 years old [1]. A meta-analysis of survey data in sub-Saharan Africa (SSA) reported that IPV was widespread in Africa, with more than a third of women (41.3%) reporting IPV during their lifetime [2]. Violence against women has received international recognition as a serious phenomenon affecting women’s lives and health, and a violation of their rights and calls for its elimination have been led by concerned organizations for decades, particularly the Sustainable Development Goal (SDG) five target 5.2 calls for the elimination of all forms of violence against all women and girls.

Violence against women by men appears to be an acceptable practice in many countries, including Nigeria [3, 4]. Nevertheless, it comes with some devastating effects on the physical and mental health and wellbeing of women, which may, in turn, influence their dietary practices, physical activity, and care for their children [5, 6]. It has also been linked to some adverse reproductive consequences among women, including non-use of antenatal care and contraception, unwanted pregnancy or mistimed pregnancy, and high rates of pregnancy loss, particularly induced abortions, miscarriages, stillbirths, and low birthweight [7–10]. The consequences of exposure to IPV extends beyond the victim. Studies have found that children who grow up in families with such violence become victims or perpetrators of domestic violence [11–13]. There is also evidence linking women’s lifetime exposure to IPV to children’s nutritional indicators, although the relationship has been inconsistent and varied by context [14–16]. Other reports even associate IPV with an increased risk of under-five mortality [17].

There is a paucity of information on the relationship between exposure to IPV and the nutritional status of women and their children under five in Nigeria. The existing studies on IPV in Nigeria have largely examined the experiences or women’s attitudes towards violence and/or their determinants [12, 18–20]. Besides the focus, these studies are also limited by the small sample size used, which weakens their generalizability. The population-based studies do not also provide any information on the relationship between exposure to IPV and women’s and children’s nutritional outcomes. For example, Ononokpono et al, examined how exposure to IPV is associated with the use of maternal health care services [21]. Antai & Antai assessed rural women’s attitudes toward IPV as well as the determinants [4]. Similarly, the study by Okenwa-Emegwa and his counterparts examined attitudes toward physical IPV against women, although their sample somewhat differed from that of Antai & Antai because it included men aged 15–49 years [3]. Finally, the study by Benebo et al examined the effect of individual- and community-level factors on IPV, with a focus on women’s status and community-level norms among men [22]. As a result, the association between women’s IPV exposure and nutritional status in Nigeria is unclear.

The purpose of the current study is to evaluate the relationship between IPV and the nutritional status of women and their children in Nigeria. We relied on a nationally representative sample from the 2018 Nigeria Demographic and Health Survey to achieve the aim of this study.

## Methods

### Theoretical framework

This current study relied on existing literature to articulate the relationship between exposure to IPV and women’s and children’s nutritional status. IPV exposure can cause a variety of health issues in women, including stress-induced psychological changes. When compared to their non-abused peers, abused women have higher rates of mental disorders such as depression, anxiety, and memory loss [23–26]. Stress has been shown to have a bidirectional effect on food intake, causing either an increase or a decrease [27]. Several studies have identified biological pathways that mediate the relationship between stress and feeding behaviour, as well as body weight [28–30]. On the one hand, most studies have found that stress, particularly chronic stress, causes weight loss by increasing metabolic rate and energy expenditure [31, 32]. On the other hand, the phenomenon of stress induced positive energy intake has been associated with an increased risk of developing obesity. Long-term stress, according to Bjorntorp, causes prolonged hyperactivation of the hypothalamo-pituitary-adrenal (HPA) axis, which increases circulating glucocorticoids that bind to glucocorticoid receptors (GR) that are highly expressed in abdominal fat, activating lipoprotein lipase and inhibiting lipid mobilization in the presence of insulin. Triglyceride levels rise as a result, as does abdominal fat retention [33].

The relationship between IPV exposure in women and children’s nutritional status may occur in utero or indirectly through effects on other family processes. Children rely heavily on maternal care. As a result, a mother’s exposure to domestic violence may have an impact on her child’s nutritional status by impairing her parenting abilities. For example, the psychological and physical effects of IPV affect women’s breastfeeding behaviour [34, 35]. Furthermore, IPV exposure in women increases children’s susceptibility to undernutrition by lowering the minimum dietary intake [36]. According to the findings of reviews, witnessing IPV can also have a negative impact on the normal development of children in the family [37]. However, not all children are affected in the same way by the negative nutritional outcomes associated with their mothers’ IPV exposure. Indeed, other pathways, in addition to maternal behaviours, have been linked to children’s nutritional outcomes. Maternal nutritional status has been shown to influence their children’s nutritional status [38–40]. Obese women, in particular, were less likely to have undernourished children, possibly due to shared genes [41, 42].

In conclusion, we contend that IPV exposure can affect women’s nutritional status through stress-induced psychological distress, which can increase or decrease food intake as well as influence metabolic rate. Furthermore, women’s poor mental health as a result of IPV exposure may impair their ability to care for children, including feeding and health seeking behaviour, both of which are predictors of children’s nutritional outcomes.

### Study design and data

This cross-sectional study analyzed secondary data obtained from the 2018 Nigeria Demographic and Health Survey (2018 NDHS). Briefly, the survey was the sixth survey of its kind to be implemented by the National Population Commission (NPC). The sample for the 2018 NDHS was chosen using a stratified, two-stage cluster design, with enumeration areas (EAs) serving as sampling units in the first stage. The second stage involved a complete listing of households in each of the 1,400 selected EAs. Women between the ages of 15 and 49 and men between the ages of 15 and 59 were targeted in randomly selected households across Nigeria. The survey used a representative sample of approximately 42,000 households. In the subsample of households chosen for the men’s survey, one eligible woman was chosen at random from each household to answer additional questions about domestic violence. The nutritional status of women and children in these households was also assessed (based on weight and height measurements). More information about the 2018 NDHS, including response rates and questionnaires used for the survey, can be found in the final report [43].

The individual and children’s recode datasets were combined for this investigation. Women aged 15 to 49 years and children aged 6 to 59 months were both studied. The study only included women who were currently in a union (married or living with a partner) and had data on the factors used to evaluate domestic violence, as well as their weight (kg) and height (cm), which were used to calculate the Body Mass Index (BMI). After removing women who were not in a union, women who were now pregnant, observations with incomplete data on IPV, and women’s weight and height, the study included a total of 4,683 women. Children without z-scores and children whose parents lacked data on IPV indicators were also eliminated. A total of 2,425 children were included for analysis. The samples were weighted during the analysis.

### Variables

#### Dependent variables

Two groups of dependent variables were studied. The first was women’s nutritional status, as determined by the BMI. The BMI was calculated by dividing the weight (kg) of the woman by her height (m^2^). The BMI cut-off used by the World Health Organization (WHO) was used to define women’s nutritional status. Thus, underweight was defined as a BMI < 18.5kg/m^2^, normal was defined as BMI of 18.5–24.9 kg/m^2^, and BMI ≥ 25 kg/m^2^ was used to define overweight/obese [44]. We recognize that the BMI is the most useful, though rather crude, population-level measure of nutritional status. It can be used to estimate the prevalence of underweight, overweight, and obesity within a population, as well as the risk factors. However, BMI does not account for the wide variation in body fat distribution, and may not correspond to the same degree of fatness in individuals and populations [45]. Nevertheless, it is the most commonly used indictor for measuring chronic energy deficiency in adults and has been used extensively in several epidemiological studies [46–48].

The second group of dependent variables was the nutritional status of children, measured by three outcomes: stunting (height-for-age; HAZ), underweight (weight-for-age; WAZ) and wasting (weight-for-height; WHZ). The WHO growth standards were used as a reference to classify children as being stunted, underweight, or wasted, respectively [49]. Accordingly, stunting, underweight, and wasting were defined by z-scores of less than -2 standard deviations (SD) from the median for HAZ, WAZ, and WHZ, respectively.

#### Main predictor variable

The primary exposure variable, IPV, was measured based on 13 questions related to physical intimate partner violence (push, shake, or throw something; slap or twist arm; punch with a fist or something that could hurt; kick or drag; try to strangle or burn; threaten with a knife, gun, or other weapon; attack with a knife, gun, or other weapon), psychological intimate partner violence (humiliated in front of others; threatened or had someone close to her threatened with harm, or insulted or made to feel bad about herself), and sexual intimate partner violence (physically forced to have sexual intercourse when not wanted) committed against a woman by her current or last husband ever and in the prior year. From this information, three binary variables were created to measure IPV against women, including psychological IPV, sexual IPV, and physical IPV. Women with previous experience of IPV were coded “1”, and those who reported never experiencing any IPV were coded “0”. A final composite IPV variable was generated and dichotomized: whether the currently married woman reported previous experience of any kind of IPV in her lifetime or the past 12 months or perpetrated by her partner (coded “1”) or never (coded “0”) (see S1 Table).

#### Confounding factors

Several covariates, including maternal age, education, place of residence, number of children ever born, frequency of watching television, current use of contraception, wealth quintile, and household cooking fuel, were included as potential confounders. According to the existing literature, these covariates have been shown to predict the nutritional status of women [50–54]. Factors included as covariates in assessing children’s nutritional status included child’s age in months, sex, birth order number, birthweight, diarrhoea in the past two weeks, mother’s age group, mother’s highest education, place of residence, mother’s BMI, and wealth quintile. These factors have a known association with the stunting, underweight, and wasting status of children under five years old [55–58]. We acknowledge that the factors used as covariates in this study are not all-inclusive. However, we were only able to include the factors found in the DHS dataset.

### Statistical analysis

Descriptive statistics were carried out to understand the distribution of study participants by the key explanatory variable, covariates, and outcome variables. Bivariate percentage distribution was estimated to assess the prevalence of IPV and children’s nutritional indicators (stunting, underweight, and wasting) by the explanatory variables, and the differences were tested by Pearson’s Chi-square test. The sample weight was used for the estimation of the percentage distribution. Finally, a series of regression models were employed to examine the association between women’s experience of IPV and their nutritional status as well as their children’s nutritional status. First, an adjusted association was estimated between the IPV and the BMI of mothers using multinomial regression, with normal weight as the base outcome. The results were presented by the estimated adjusted relative risk ratio (ARRR). Second, adjusted binary logistic regression models were used for each child nutritional indicator to examine the association between maternal experience of IPV and the nutritional status of children (stunting, underweight, and wasting). The regression results were presented as the estimated adjusted odds ratio (AOR) with a 95% confidence interval (CI). The significance level for regression analyses was set at p < 0.05. All the statistical analyses were performed using STATA version 13.0 (StataCorp LP, College Station, TX, USA).

### Ethical consideration

This study is based on secondary information that is already available in the public domain. Therefore, ethical approval was not required for this study. However, the ethical procedures followed by the DHS program in its surveys are published online at https://dhsprogram.com/Methodology/Protecting-the-Privacy-of-DHS-Survey-Respondents.cfm

## Results

### Prevalence and distribution of intimate partner violence by women’s characteristics

The prevalence of the three types of IPV by women’s characteristics is shown in Table 1. Among the 4391 women studied, 6.03% (95% CI: 5.12, 7.09) experienced sexual IPV, 30.43% (95% CI: 28.54, 32.38) experienced psychological IPV, and 19.43% (95% CI: 17.79, 21.19) experienced physical IPV. In total, 35.31% (95% CI: 33.35, 37.33) of women have experienced at least one form of IPV in their lifetime.

**Table 1:** Prevalence of types of intimate partner violence by women’s characteristics (weighted N = 4,391).

Characteristic	Weighted	Sexual IPV	Psychological IPV	Physical IPV	Any form of IPV
	%	% (95% CI)	% (95% CI)	% (95% CI)	% (95% CI)
**All women**		6.03(5.12, 7.09)	30.43(28.54, 32.38)	19.43(17.79, 21.19)	35.31(33.35, 37.33)
**Age group (years)**					
15–24	11.52	8.56(5.53, 13.04)	26.74(21.81, 32.33)	14.65(11.34, 18.72)	32.33(27.29, 37.81)
25–34	37.78	6.14(4.73, 7.93)	30.37(27.2, 33.63)	20.03(17.58, 22.73)	34.69(31.50, 38.02)
35–49	50.70	5.37(4.34, 6.64)	31.30(28.8, 33.92)	20.08(17.84, 22.51)	36.45(33.83, 39.16)
p-value		0.138	0.333	0.065	0.364
**Place of residence**					
Rural	41.89	6.81(5.58, 8.30)	33.75(31.13, 36.47)	20.09(17.99, 22.36)	38.96(36.30, 41.69)
Urban	58.11	5.47(4.24, 7.02)	28.03(25.45, 30.76)	18.96(16.62, 21.55)	32.68(29.95, 35.53)
p-value		0.177	0.003	0.504	0.002
**Religious affiliation**					
African traditional	0.44	4.34(1.18, 14.77)	36.61(19.45, 58.00)	27.09(12.33, 49.54)	40.55(22.44, 61.67)
Islam	36.78	5.23(3.94, 6.90)	22.54(19.50, 25.92)	11.98(9.69, 14.71)	25.97(22.79, 29.42)
Christian	62.78	6.51(5.31, 7.97)	35.00(32.57, 37.50)	23.75(21.52, 26.13)	40.75(38.2, 43.35)
p-value		0.258	<0.001	<0.001	<0.001
**Number of children ever born**					
1–3	53.30	6.23(4.92, 7.85)	27.72(25.27, 30.32)	18.04(16.00, 20.28)	32.17(29.55, 34.89)
4 or more	46.70	5.81(4.73, 7.11)	33.51(30.72, 36.42)	21.02(18.74, 23.50)	38.90(36.02, 41.87)
p-value		0.646	0.003	0.048	0.001
**Highest education**					
No formal education	23.12	5.88(4.28, 8.02)	30.32(26.24, 34.74)	16.46(13.13, 20.44)	33.80(29.48, 38.41)
Primary	16.63	6.15(4.35, 8.63)	32.88(28.46, 37.61)	23.26(19.42, 27.62)	37.74(32.96, 42.78)
At least secondary	60.25	6.05(4.83, 7.56)	29.79(27.35, 32.35)	19.52(17.50, 21.70)	35.22(32.71, 37.82)
p-value		0.981	0.533	0.049	0.506
**Current use of contraception**					
Not using	70.68	6.11(5.162, 7.22)	30.32(28.16, 32.58)	18.74(16.91, 20.72)	34.76(32.53, 37.07)
Currently using	29.32	5.84(3.95, 8.56)	30.68(27.02, 34.6)	21.10(18.01, 24.57)	36.63(32.77, 40.68)
p-value		0.835	0.873	0.197	0.418
**Frequency of watching television**					
Not at all	32.35	7.95(6.23, 10.08)	35.13(31.65, 38.77)	19.64(16.91, 22.69)	38.74(35.23, 42.38)
Less than once a week	22.03	4.83(3.33, 6.96)	27.47(23.67, 31.63)	18.84(15.68, 22.46)	32.23(28.21, 36.54)
At least once a week	45.62	5.25(4.09, 6.71)	28.52(25.77, 31.44)	19.57(17.29, 22.07)	34.37(31.21, 36.54)
p-value		0.017	0.004	0.924	0.049
**Wealth quintile**					
Poorest	9.32	7.88(5.59, 10.98)	29.54(23.95, 35.82)	14.65(10.90, 19.41)	33.23(27.52, 39.47)
Poorer	12.21	7.77(5.33, 11.19)	39.26(33.72, 45.09)	22.35(17.69, 27.83)	42.47(36.96, 48.18)
Middle	16.97	7.38(5.12, 9.80)	35.09(30.94, 39.48)	21.64(18.42, 25.25)	39.49(35.23, 43.91)
Richer	24.46	7.19(4.92, 10.38)	33.82(29.46, 38.48)	24.20(20.73, 28.06)	40.14(35.47, 45.00)
Richest	37.04	3.61(2.56, 5.08)	23.35(20.31, 26.70)	15.51(13.00, 18.41)	28.37(25.17, 31.82)
p-value		0.005	<0.001	<0.001	<0.001
**Household cooking fuel**					
Clean fuel	41.67	5.05(3.70, 6.85)	25.97(23.04, 29.14)	17.79(15.33, 20.55)	31.32(28.14, 34.70)
Solid fuel	58.33	6.80(5.61, 8.20)	33.31(30.86, 35.84)	20.04(17.95, 22.31)	37.79(35.29, 40.36)
p-value		0.104	0.001	0.205	0.003
**Nutritional status of woman**					
Normal	49.17	6.88(5.60, 8.41)	29.97(27.33, 32.75)	20.29(18.06, 22.71)	34.30(31.57, 37.15)
Underweight	5.71	4.96(2.53, 9.49)	21.22(15.88, 27.76)	15.06(10.51, 21.13)	25.75(19.95, 32.55)
Overweight/Obese	45.12	5.35(4.12, 6.90)	32.57(29.61, 35.68)	19.33(17.07, 21.80)	38.26(35.19, 41.43)
p-value		0.216	0.018	0.265	0.006

The results also showed that women who experienced IPV were similar to those who did not on some characteristics but differed on others. For instance, on one hand, women who experienced sexual IPV differed significantly from those who did not in terms of frequency of watching television and wealth quintile. On the other hand, women who experienced physical IPV were similar to those who did not experience physical IPV in terms of age, place of residence, current use of contraception, frequency of watching television, type of household cooking fuel, and nutritional status. The prevalence of overweight/obesity was higher for women who experienced psychological IPV (p = 0.018). Similarly, women who experienced at least one form of IPV were significantly more likely to be overweight or/obese than those who did not experience any violence (Table 1).

### Association between women’s exposure to intimate partner violence and their nutritional status

The multinomial regression showed that women who experienced any form of IPV in their lifetime were less likely to be underweight (ARRR = 0.63; 95% CI: 0.44, 0.91) and more likely to be overweight/obese (ARRR = 1.28; 95% CI: 1.04, 1.58) after adjusting for women’s demographic and socioeconomic factors. The results also demonstrated that age, number of children ever born, education, frequency of watching television, and wealth quintile were associated with women’s nutritional status (Table 2).

**Table 2:** Association between exposure to intimate partner violence and women’s nutritional status (weighted N = 4,391).

Characteristic	Base outcome (Normal weight)			
	Underweight		Overweight/Obesity	
	ARRR (95% CI)	p-value	ARRR (95% CI)	p-value
**Any form of IPV**				
Never (Ref)	1.00		1.00	
Yes	0.63(0.44, 0.91)	0.013	1.28(1.04,1.58)	0.018
**Age group (years)**				
15–24 (Ref)	1.00		1.00	
25–34	1.88(1.21, 2.92)	0.005	1.91(1.33, 2.75)	0.001
35–49	1.45(0.91, 2.32)	0.121	3.10((2.13, 4.50)	<0.001
**Place of residence**				
Rural (Ref)	1.00		1.00	
Urban	0.62(0.42, 0.93)	0.021	1.09(0.89, 1.34)	0.442
**Religious affiliation**				
African traditional (Ref)	1.00		1.00	
Islam	0.98(0.19, 4.94)	0.979	1.01(0.34, 2.95)	0.991
Christian	0.65(0.13, 3.40)	0.614	1.53(0.53, 4.47)	0.433
**Number of children ever born**				
1–3 (Ref)	1.00		1.00	
4 or more	0.58(0.40, 0.83)	0.003	1.47(1.18, 1.83)	<0.001
**Highest education**				
No formal education (Ref)	1.00		1.00	
Primary	0.47(0.27, 0.82)	0.008	0.81(0.57, 1.16)	0.252
At least secondary	0.68(0.42, 1.10)	0.114	0.92(0.65, 1.31)	0.660
**Current use of contraception**				
Not using (Ref)	1.00		1.00	
Currently using	1.04(0.67, 1.60)	0.862	1.19(0.94, 1.51)	0.154
**Frequency of watching television**				
Not at all (Ref)	1.00		1.00	
Less than once a week	0.79(0.50, 1.23)	0.294	1.32(0.97, 1.81)	0.082
At least once a week	1.28(0.80, 2.03)	0.300	1.61(1.19, 2.19)	0.002
**Wealth quintile**				
Poorest (Ref)	1.00		1.00	
Poorer	0.81(0.47, 1.37)	0.432	1.40(0.88, 2.21)	151.000
Middle	0.78(0.46, 1.32)	0.348	2.56(1.66, 3.96)	<0.001
Richer	0.55(0.29, 1.04)	0.066	3.56(2.17, 5.84)	<0.001
Richest	0.23(0.10, 0.49)	<0.001	5.38(3.03, 9.55)	<0.001
**Household cooking fuel**				
Clean fuel (Ref)	1.00		1.00	
Solid fuel	0.63(0.35, 1.14)	0.129	0.86(0.63, 1.19)	0.372

Ref: Reference group.

### Prevalence of childhood undernutrition among children of women with a lifetime exposure to intimate partner violence

**Fig 1:**
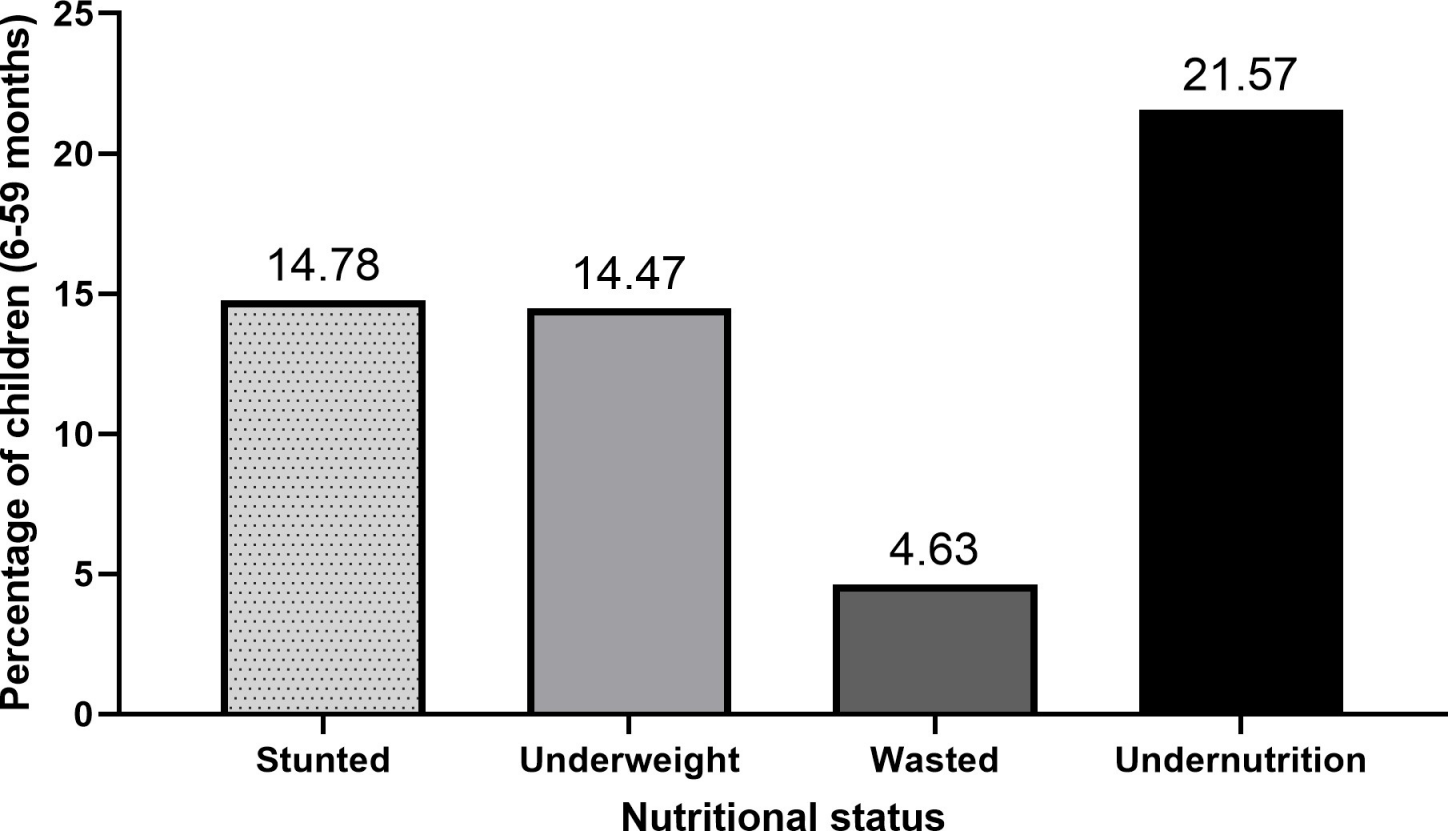
Distribution of undernutrition among children (6–59 months) of women with a lifetime exposure to violence by an intimate partner (N = 2145).

The results revealed that of the 2145 children analysed, 14.78% (95% CI: 12.85–16.95) were stunted, 14.47% (95% CI: 12.72–16.41) were underweight, and 4.63% (95% CI: 3.56–6.01) were wasted according to the WHO classifications. Overall, 21.57% (95% CI: 19.32–24.01) of the children had at least one form of childhood undernutrition (Fig 1).

### Distribution of undernutrition among children aged 6–59 months by mother’s exposure to intimate partner violence and child’s characteristics

The distribution of undernutrition among children 6–59 months by mother’s exposure to IPV and child’s characteristics is presented in Table 3. According to the results, among the three nutrition indicators, only the prevalence of underweight varied widely among children with respect to mother’s lifetime exposure to IPV. Regarding child’s characteristics, the three nutrition indicators varied by child’s age, stunting and underweight varied significantly by birthweight and diarrhoea in the past 2 weeks (Table 3).

**Table 3:** Distribution of undernutrition among children aged 6–59 months by exposure to intimate partner violence and child’s characteristics (weighted N = 2,145).

Characteristic	Weighted	Stunted	Underweight	Wasted
	%	%(95% CI)	%(95% CI)	%(95% CI)
**All children**		14.78(12.85–16.95)	14.47(12.72–16.41)	4.63(3.56–6.01)
**Any form of IPV**				
Never	65.63	14.66(12.4, 17.25)	15.87(13.57, 18.48)	5.12(3.68, 7.09)
Yes	34.37	15.01(11.76, 18.96)	11.79(9.49, 14.57)	3.69(2.53, 5.37)
p-value		0.873	0.026	0.196
**Child factors**				
**Age (months)**				
6–11	12.76	10.62(6.88, 16.04)	17.36(12.73, 23.23)	5.39(3.19, 8.968)
12–23	25.74	19.08(14.85, 24.18)	21.48(16.99, 26.77)	10.22(6.82, 15.02)
24–59	61.50	13.84(11.52, 16.55)	10.93(9.15, 13.00)	2.14(1.48, 3.09)
p-value		0.026	<0.001	<0.001
**Sex**				
Male	52.22	17.71(14.75, 21.11)	16.07(13.53, 18.98)	5.64(3.91, 8.08)
Female	47.78	11.58(9.53, 14.00)	12.71(10.57, 15.21)	3.53(2.51, 4.94)
p-value		0.001	0.058	0.064
**Birth order number**				
1	26.68	11.04(8.64, 14.00)	12.02(9.26, 15.47)	4.60(3.07, 6.85)
2	24.03	15.69(11.72, 20.70)	14.37(10.52, 19.31)	5.20(2.48, 10.60)
3	18.00	13.60(10.34, 17.69)	15.34(11.74, 19.80)	5.65(3.66, 8.62)
4+	31.29	17.94(13.99, 22.72)	16.13(13.06, 19.75)	3.64(2.34, 5.61)
p-value		0.051	0.398	0.641
**Birthweight**				
Normal	93.70	13.55(11.76, 15.55)	13.64(11.92, 15.57)	4.57(3.47, 5.98)
Low birthweight	6.30	33.15(21.93, 46.68)	26.71(17.8, 38.02)	5.63(2.21, 13.61)
p-value		<0.001	0.001	0.665
**Diarrhoea in the past 2 weeks**				
No	93.27	13.97(11.98, 16.23)	13.44(11.69, 15.41)	4.57(3.47, 6.01)
Yes	6.74	25.94(18.92, 34.47)	28.73(21.20, 37.65)	5.48(2.78, 10.51)
p-value		0.001	<0.001	0.614

### Association between women’s experiences with intimate partner violence and their children’s nutritional status

In Table 4, the association between women’s experience of IPV and children’s nutritional status is presented. The data showed that, on one hand, women’s experience of IPV was not significantly associated with stunting and wasting. On the other hand, an association existed between IPV and underweight; children of mothers who experienced IPV were less likely to be underweight compared to those whose mothers did not experience IPV after adjusting for confounding effects by the child and maternal-related factors. We also found that children of mothers who were classified as overweight or obese were 32% and 45% less likely to be stunted or underweight, respectively (Table 4).

**Table 4:** Association between women’s experience with intimate partner violence and their children’s nutritional status (weighted N = 2,142).

Characteristic	Stunted AOR (95% CI)	p-value	Underweight AOR (95% CI)	p-value	Wasted AOR (95% CI)	p-value
**Any form of IPV**						
Never (Ref)	1.00		1.00		1.00	
Yes	0.94(0.68, 1.30)	0.703	0.69(0.50, 0.96)	0.025	0.83(0.52, 1.35)	0.467
**Child factors**						
**Age (months)**						
6–11 (Ref)	1.00		1.00		1.00	
12–23	2.14(1.18, 3.91)	0.013	1.27(0.79, 2.03)	0.330	2.01(1.03, 3.89)	0.040
24–59	1.56(0.89, 2.74)	0.119	0.61(0.39, 0.96)	0.031	0.39(0.19, 0.79)	0.009
**Sex**						
Male (Ref)	1.00		1.00		1.00	
Female	0.61(0.46, 0.83)	0.001	0.79(0.59, 1.04)	0.097	0.67(0.42, 1.06)	0.085
**Birth order number**						
1 (Ref)	1.00		1.00		1.00	
2	1.84(1.21, 2.81)	0.005	1.25(0.82, 1.92)	0.299	1.11(0.56, 2.21)	0.770
3	1.63(1.02, 2.61)	0.041	1.34(0.84, 2.24)	0.204	1.24(0.59, 2.62)	0.567
4+	2.16(1.22, 3.84)	0.009	1.45(0.85, 2.46)	0.175	0.71(0.27, 1.90)	0.500
**Birthweight**						
Normal (Ref)	1.00		1.00		1.00	
Low birthweight	2.78(1.60, 4.81)	<0.001	2.07(1.23, 3.48)	0.006	1.19(0.44, 3.22)	0.729
**Diarrhoea in the past 2 weeks**						
No (Ref)	1.00		1.00		1.00	
Yes	1.86(1.14, 3.05)	0.014	2.26(1.43, 3.59)	0.001	0.89(0.40, 1.92)	0.746
**Mother’s characteristics**						
**Age (years)**						
15–24 (Ref)	1.00		1.00		1.00	
25–34	0.45(0.29, 0.70)	<0.001	0.72(0.47, 1.11)	0.141	0.66(0.32, 1.36)	0.260
35–49	0.47(0.25, 0.91)	0.026	0.99(0.53, 1.82)	0.963	1.16(0.38, 3.56)	0.798
**Educational level**						
No formal education (Ref)	1.00		1.00		1.00	
Primary	0.63(0.33, 1.20)	0.160	1.02(0.52, 1.97)	0.963	2.03(0.56, 7.32)	0.278
At least secondary	0.40(0.22, 0.73)	0.003	0.65(0.35, 1.19)	0.164	1.13(0.37, 3.49)	0.826
**Place of residence**						
Urban (Ref)	1.00		1.00			
Rural	1.00(0.67, 1.49)	0.991	1.03(0.76, 1.40)	0.843	1.28(0.72, 2.30)	0.401
**Mother’s BMI**						
Normal (Ref)	1.00		1.00		1.00	
Underweight	1.32(0.78, 2.24)	0.305	1.65(0.96, 2.82)	0.068	1.83(0.92, 3.63)	0.084
Overweight/obese	0.68(0.49, 0.94)	0.023	0.55(0.39, 0.78)	0.001	0.69(0.36, 1.33)	0.266
**Wealth group**						
Poorest (Ref)	1.00		1.00		1.00	
Poorer	1.08(0.48, 2.43)	0.858	0.95(0.42, 2.15)	0.896	1.12(0.26, 4.74)	0.877
Middle	0.91(0.42, 1.99)	0.818	0.76(0.35, 1.65)	0.495	1.23(0.36, 4.18)	0.738
Richer	0.90(0.42, 1.95)	0.795	0.86(0.41, 1.80)	0.692	1.07(0.28, 4.18)	0.918
Richest	0.61(0.26, 1.40)	0.240	0.78(0.37, 1.67)	0.522	1.44(0.39, 5.36)	0.582

Ref: Reference group.

## Discussion

This study examined the relationship between exposure to IPV and women’s and child nutritional outcomes in Nigeria using a nationally representative dataset. The study found that the majority (30.4%) of women experienced psychological IPV, followed by physical IPV (19.4%), and sexual IPV (6.0%). In total, more than a third of the women experienced at least one form of IPV in their lifetime. Another study conducted in Nigeria reported results that are similar to those of the present study [22]. Other studies in Nigeria reported relatively higher rates of IPV among women. For example, the study by Arulogun et al. found that 38.0% of women experienced psychological IPV, while 36.4% experienced physical IPV [18]. We believe that patriarchy, social and cultural gender norms, power dynamics, and hierarchical constructions of masculinity and womanliness are the driving forces behind IPV in the study setting [3, 4, 22]. Findings from other parts of the world, particularly Asia, show a similar trend. A study in Bangladesh discovered that 14.5% and 29.0% of partnered women experienced sexual and physical IPV, respectively [59]. Similarly, a study discovered that 23.0%, 12.2%, and 7.0% of ever-married Nepalese women experienced physical, emotional, and sexual IPV, respectively [50]. In this context, the data suggest that psychological and physical violence against women by their spouse/partner are more common than sexual violence. Despite this, victims of domestic violence were more likely to seek help from family members and friends rather than reporting the incident to authorities or other anti-violence organizations [12].

The study found that a significant percentage of women who experienced psychological IPV and at least one form of IPV were overweight or obese compared to those who did not. The multinomial regression analysis provided evidence of the strength of this relationship, with women with a lifetime exposure to IPV having a 63.0% reduced risk of being underweight and a 28.0% increased risk of overweight or obesity. A population-based study from Bangladesh established that exposure to IPV was associated with an increased risk of overweight and obesity [59]. Similar findings were noted among Egyptian women [51]. Some studies, however, found that IPV increased the risk of being underweight among women in low-income settings, which contradicts our findings [60, 61]. We recognize that violence against women can lead to poor mental health and chronic stress, which increases the secretion of glucocorticoids and insulin, influencing eating behaviour by either decreasing or increasing food intake [27]. Chronic stress has been linked to weight loss by increasing metabolic rate and energy expenditure [31, 32]. However, studies have also documented a link between prolonged chronic stress and obesity, mediated by metabolic changes and behavioural adjustments, which promote the deposition of abdominal adipose tissue [62, 63]. As a result, it is plausible to conclude that women in this study who had IPV may have psychological problems and hormonal changes, which increase their appetite and cause a craving for food, particularly energy-dense foods, which are common in Nigeria, increasing their risk of being overweight or obese [64]. This claim is backed by evidence from animal models, which show that stress increases the consumption of high-fat and high-carbohydrate diets [65].

Nevertheless, the association of IPV with overweight and obesity may also hamper the global control of obesity. Overweight among women has increased throughout the world since 1990. In SSA, the prevalence of overweight increased from 16% in 1990 to 22% in 2010 [66]. The global increases in overweight and obesity appear to be driven more by domestic processes, including economic development, urbanization, and women’s empowerment and are less clearly negatively impacted by external globalization processes, suggesting that the harm to health from global trade regimes may be overstated [67]. However, it is also possible that IPV is to blame for women’s weight gain in LMICs, particularly in countries with a high prevalence of IPV.

The study found that 21.6% of the children had at least one form of childhood undernutrition. Specifically, 14.8% of the children were stunted, 14.5% were underweight, and 4.6% were wasted. These rates are lower compared to what has been reported elsewhere [68]. An earlier study discovered that women’s exposure to IPV compromised child growth; particularly, IPV was significantly related to the risk of stunting among children [15]. A pooled analysis of DHS data from 29 countries showed a positive association between stunting in children and maternal lifetime exposure to IPV and a small negative association between wasting and IPV [16]. In other LMICs, maternal reports of physical or sexual IPV predicted higher odds of stunting and being underweight [69, 70]. The findings of our study, however, indicate otherwise; children of mothers who experienced IPV were less likely to be underweight. Mothers not only create the food environment for their children at home, but also influence their eating behaviours, taste preferences, and food choices [71]. Accordingly, the physical and psychological problems induced by IPV can reduce a mother’s caregiving abilities. A study from Ethiopia found that IPV decreases children’s minimum acceptable diet intake by 65%, increasing their susceptibility to undernutrition [36]. Nevertheless, existing evidence suggests that other pathways beyond maternal behaviours are closely associated with child health outcomes [42]. Maternal obesity confers the risk of obesity on children through shared genes [41, 42]. Moreover, there is evidence of a direct link between mother and child anthropometric indices, as shown in this study and other documentation [38–40]. Although our study did not assess overweight and obesity among children, it is conceivable that genetic predispositions contributed to reducing the risk of being underweight among children whose parents are overweight or obese and have been exposed to IPV.

The study found that other factors aside from IPV were significantly associated with maternal and child nutritional status. There are existing reports for and against the relationship of these factors with maternal BMI [52, 54, 72] and childhood undernutrition [55, 68, 73–75]. In Ghana, for example, obesity and overweight were found to be more common among older women, urban women, married women, women with higher education, and women from rich households [52, 76]. In Ethiopia, higher odds of stunting was found among girls than boys, which contradicts our findings [55]. A review identified several factors, which predispose children to childhood stunting, wasting, and underweight in SSA, some of which have been identified in our study, including low mother’s education, increasing child’s age, sex of child (male), low birth weight, mother’s age, low mother’s BMI (<18.5), birth size (small), and diarrhoeal episode [57].

### Strengths and limitations

The study made use of nationally representative data with a large sample size, which strengthens the external validity. The study also had some limitations that needed to be considered in the interpretation of the results. The study design precludes us from drawing causal inferences from the results obtained. There is also the possibility that the prevalence of IPV against women in the population was underestimated due to underreporting. Finally, we acknowledge that the BMI is a crude way of measuring nutritional status because it does not account for variation in body fat distribution. Nonetheless, among adults, it is the most commonly used measure of nutritional status. The covariates used in this study, on the other hand, are not all-inclusive and were limited to those found in the DHS dataset.

## Conclusion

Overall, IPV against women, particularly psychological, physical, and sexual IPV, is common in Nigeria and has an association with the nutritional status of affected women and their children. According to the study, women with a lifetime experience of IPV were more likely to be overweight and affected women’s children were less likely to be underweight. A far-reaching effort is required to curb IPV against women, particularly policies, programs, and laws are needed to protect women and children from the unfavourable effects of IPV to reduce the prevalence and impact of such violence. In Nigeria, patriarchy and social and cultural gender norms are predominant and pervasive drivers of IPV. As a result, strategies for changing these norms are required. The findings from this study may help the national government, non-governmental and civil society organizations in incorporating issues pertaining to intimate partner violence in general, as well as public health policies aimed at preventing the occurrence of violence, in order to achieve target 5.2 of Goal 5 of the Sustainable Development Goals, which talks about eliminating all forms of violence against females.

## Supporting information

S1 Table Women’s responses to questions on intimate partner violence. (DOCX)

## Abbreviations

ARRR: Adjusted Relative Risk Ratio
AOR: Adjusted Odds Ratio
BMI: Body Mass Index
GR: glucocorticoid receptors
HPA: hypothalamo-pituitary-adrenal axis
IPV: Intimate Partner Violence
NDHS: Nigeria Demographic and Health Survey
NPC: National Population Commission
SSA: sub-Saharan Africa
SD: Standard Deviation
SDGs: Sustainable Development Goals
WHO: World Health Organization
