# Supplementary material for: Association between exposure to intimate partner violence and the nutritional status of women and children in Nigeria
Source: PLoS One. 2022 May 12;17(5):e0268462. doi: 10.1371/journal.pone.0268462 (PMC9098093; doi:10.1371/journal.pone.0268462)
Supplement: S1 Table — (DOCX) [file pone.0268462.s001.docx]

**S1 Table: Women’s responses to questions on a lifetime experience of intimate partner violence (IPV) (Weighted N=4,391)**

| **Questions** | **Responses: Percentage of women** | |
| --- | --- | --- |
|  | **Yes (“1”)** | **No (“0”)** |
| **Experience of physical IPV**  Did your (last) (husband/partner) ever do any of the following things to you: |  |  |
| 1. Push you, shake you, or throw something at you? | 7.59 | 92.41 |
| 1. Slapped you? | 16.58 | 83.42 |
| 1. Punch you with his fist or with something that could hurt you? | 4.86 | 95.14 |
| 1. Kick you, drag you, or beat you up? | 9.49 | 90.51 |
| 1. Try to choke you or burn you on purpose? | 0.90 | 99.10 |
| 1. Threaten or attack you with a knife, gun, or other weapon? | 1.07 | 98.93 |
| 1. Twist your arm or pull your hair? | 3.13 | 96.87 |
| **Lifetime exposure to any form of physical IPV** | **19.43** | **80.57** |
|  |  |  |
| **Experience of psychological IPV**    Did your (last) (husband/partner) ever: |  |  |
| 1. Say or do something to humiliate you in front of others? | 17.03 | 82.97 |
| 1. Threatened to hurt or harm you or someone you care about? | 6.47 | 93.53 |
| 1. Insulted or made you feel bad about yourself? | 26.87 | 73.13 |
| **Lifetime exposure to psychological IPV** | 30.43 | 69.57 |
|  |  |  |
| **Experience of sexual IPV**    Did your (last) (husband/partner) ever do any of the following  things to you: |  |  |
| 1. Physically force you to have sexual intercourse with him when you did not want to? | 5.37 | 94.63 |
| 1. Force you with threats or in any other way to perform sexual acts you did not want to? | 1.72 | 98.28 |
| 1. Physically force you to perform any other sexual acts you did not want to? | 2.21 | 97.79 |
| **Lifetime exposure to sexual IPV** | **6.03** | **93.97** |
| **Lifetime exposure to any form of IPV** | **35.31** | **64.69** |
